# How T118M peripheral myelin protein 22 predisposes humans to Charcot–Marie–Tooth disease

**DOI:** 10.1016/j.jbc.2022.102839

**Published:** 2022-12-26

**Authors:** Katherine M. Stefanski, Geoffrey C. Li, Justin T. Marinko, Bruce D. Carter, David C. Samuels, Charles R. Sanders

**Affiliations:** 1 Department of Biochemistry, Vanderbilt University School of Medicine, Nashville, Tennessee, USA; 2 Center for Structural Biology, Vanderbilt University School of Medicine, Nashville, Tennessee, USA; 3 Department of Molecular Physiology and Biophysics, Vanderbilt University School of Medicine, Nashville, Tennessee, USA; 4 Department of Medicine, Vanderbilt University School of Medicine, Nashville, Tennessee, USA

**Keywords:** peripheral neuropathy, peripheral myelin protein 22, PMP22, Charcot–Marie–Tooth disease, hereditary neuropathy with pressure palsies, carpal tunnel syndrome, CMT, Charcot–Marie–Tooth, CMT1A, CMT type 1A, CMTE, CMT type E, CTS, carpal tunnel syndrome, DDM, dodecylmaltoside, DSS, Dejerine–Sottas syndrome, EMR, electronic medical record, HEK, human embryonic kidney cell line, HNPP, hereditary neuropathy with pressure palsy, HSQC, heteronuclear single quantum coherence, ICD, International Classification of Disease, MDCK, Madin–Darby canine kidney, Ni–NTA, nickel–nitrilotriacetic acid, PM, plasma membrane, PMP22, peripheral myelin protein 22, TCEP, Tris(2-carboxyethyl)phosphine, TROSY, transverse relaxation optimized spectroscopy

## Abstract

Data from gnomAD indicate that a missense mutation encoding the T118M variation in human peripheral myelin protein 22 (PMP22) is found in roughly one of every 75 genomes of western European lineage (1:120 in the overall human population). It is unusual among PMP22 variants that cause Charcot–Marie–Tooth (CMT) disease in that it is not 100% penetrant. Here, we conducted cellular and biophysical studies to determine why T118M PMP22 predisposes humans to CMT, but with only incomplete penetrance. We found that T118M PMP22 is prone to mistraffic but differs even from the WT protein in that increased expression levels do not result in a reduction in trafficking efficiency. Moreover, the T118M mutant exhibits a reduced tendency to form large intracellular aggregates relative to other disease mutants and even WT PMP22. NMR spectroscopy revealed that the structure and dynamics of T118M PMP22 resembled those of WT. These results show that the main consequence of T118M PMP22 in WT/T118M heterozygous individuals is a reduction in surface-trafficked PMP22, unaccompanied by formation of toxic intracellular aggregates. This explains the incomplete disease penetrance and the mild neuropathy observed for WT/T118M CMT cases. We also analyzed BioVU, a biobank linked to deidentified electronic medical records, and found a statistically robust association of the T118M mutation with the occurrence of long and/or repeated episodes of carpal tunnel syndrome. Collectively, our results illuminate the cellular effects of the T118M PMP22 variation leading to CMT disease and indicate a second disorder for which it is a risk factor.

Peripheral myelin protein 22 (PMP22) is a tetraspan membrane protein that is highly expressed in myelinating Schwann cells and abundant in peripheral nervous system myelin membranes (Fig. 1). Although the functions of PMP22 remain poorly understood, it appears to play roles in Schwann cell differentiation, cholesterol homeostasis, junction formation, modulation of the trafficking or function of other proteins, and myelin structure (1, 2, 3, 4, 5, 6, 7, 8, 9, 10, 11). That at least some of these functions are important is clear, as genetic variations impacting the *PMP22* gene result in dysmyelinating peripheral neuropathies of the Charcot–Marie–Tooth (CMT) disease family: CMT type 1A (CMT1A, caused by the presence of an extra WT *PMP22* allele because of trisomy, moderate neuropathy), CMT type E (CMTE, caused by dominant missense mutations in *PMP22*, mild to moderate neuropathy), Dejerine–Sottas syndrome (DSS, caused by dominant missense mutations in *PMP22*, severe neuropathy), and hereditary neuropathy with pressure palsies (HNPP, caused by deletion of an allele leading to PMP22 underexpression and mild neuropathy) (1, 2, 12, 13, 14). CMT is among the most common genetic diseases, affecting approximately one in 2500 people (15).

**Figure 1:**
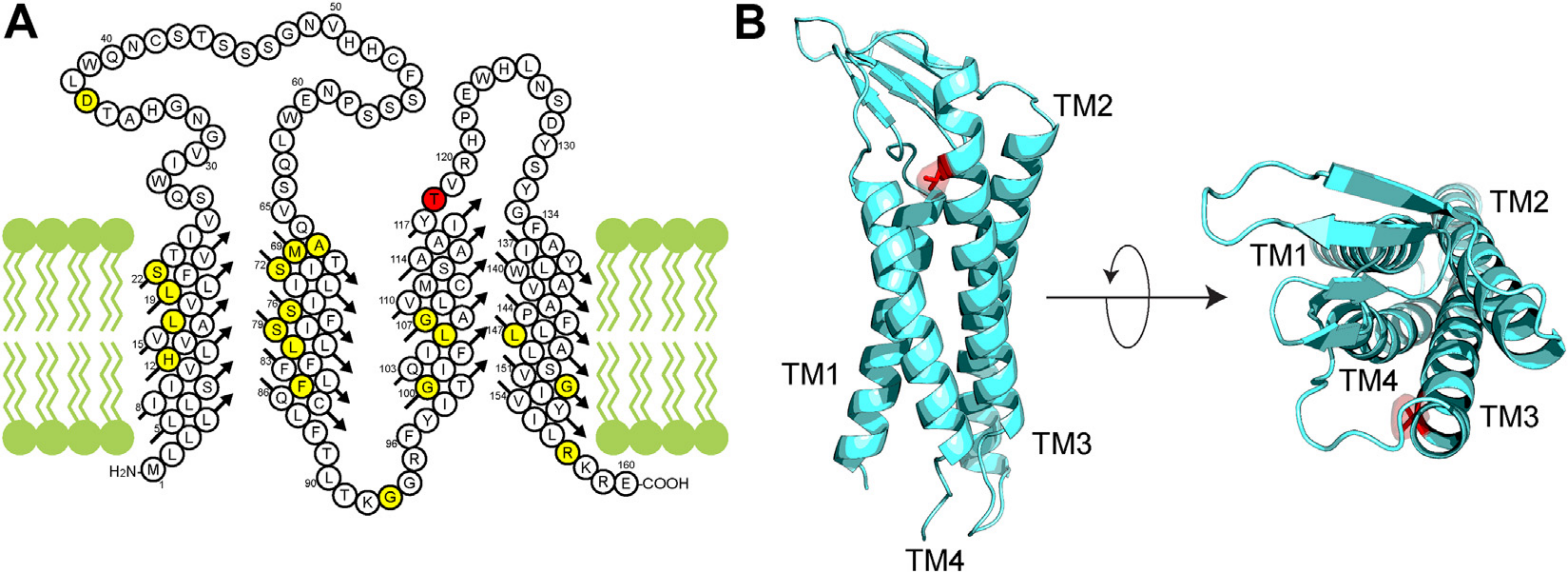
**Location of T118M site in the PMP22 protein.***A*, membrane topology diagram for the human PMP22 sequence, highlighting the location of the T118M mutation (*red*) and other known disease mutations (*yellow*). *B*, location of T118M within the alphaFold2 model of human PMP22 (48), which was seen to be almost identical to a previously published hybrid homology/ROSETTA model of human PMP22 (49). PMP22, peripheral myelin protein 22.

The ACG→ATG missense mutation in the codon of *PMP22* that encodes the T118M amino acid variation (Fig. 1) is unusual among known *PMP22* disease mutations in that it does not seem to be 100% penetrant when expressed under heterozygous WT/mutant conditions (16, 17, 18, 19). Some heterozygous carriers do not present with CMT or related peripheral neuropathies. On the other hand, some do, usually with relatively mild neuropathy. Studies of nerve conduction velocities in heterozygous human subjects have found that some individuals have moderately decreased conduction velocity, whereas others exhibit normal conduction velocities (16, 18, 19). It should be added that the very rare homozygous (T118M/T118M) individuals present with much more severe neuropathy (18) and that also-rare T118M/null patients exhibit more severe neuropathy than WT/null individuals (20). In this article, we report that the T118M PMP22 variant is common in the human population, which confirms its role as being a risk factor for CMT rather than a 100%-penetrant cause of this disorder. Cellular and biophysical studies of the T118M PMP22 and comparison to other forms of the protein reveal the defects in T118M that make it a CMT risk factor while also appearing to explain why it leads, at most, to only mild CMT disease phenotypes. We also probed a large human genetic database for which deidentified patient records are available, leading to the discovery that the T118M-encoding *PMP22* missense mutation is a risk factor for chronic or repeated incidents of carpal tunnel syndrome (CTS).

## Results

### Occurrence of the T118M PMP22–encoding mutation in the human population

The gnomAD human genome database (21) was probed for the T118M variant allele of *PMP22*. The statistics by ethnic group derived from the gnomAD 2.1 database, which represents over 125,000 people, are summarized in Table 1. The T118M variant *PMP22* allele is common in genomes of Western European lineage (1 in 75) but is less common in most other ethnic groups. For example, only one in 3300 Asians carry this allele. The incidence of the T118M *PMP22* mutation in the human population is roughly the same for women and men. Combining the data of Table 1 with US Census demographic data (https://data.census.gov/cedsci/table?tid=ACSDP5Y2016.DP05) leads to the conclusion that roughly one in every 120 people in the United States carries a T118M *PMP22* allele. An analysis of the incidence of CMT disease in the US population concluded that CMT and related peripheral neuropathies exhibit a combined prevalence of 1:2500, with about 57% of all CMT cases being CMT1A. CMT1A is caused by trisomy leading to expression of a third WT allele of *PMP22*. Another 22% of CMT patients suffer from a missing *PMP22* allele (WT/null), leading to HNPP (22). That study also showed that only about 1% of patients suffer from CMTE or the more severe DSS, both of which result from dominant missense mutations (WT/mutant) that alter the amino acid sequence of PMP22. The prevalence of these latter forms of CMT is only on the order of 1 in 250,000 in the United States, some three orders of magnitude lower than that in the 1 in 120 incidence of the T118M allele. These simple considerations lead to the conclusion that under heterozygous T118M/WT conditions, T118M PMP22 is incompletely penetrant as a cause of CMT and related neuropathies: its incidence in the US population is 1:120, but only 1:2500 in this population are known to be afflicted with any form of CMT. The penetrance of T118M PMP22 is therefore <5%. For WT/T118M individuals, the collusion of other risk factors and/or the absence of one or more normally protective factors is evidently required to cause noticeable peripheral neuropathy. We next conducted mechanistic studies to explain why heterozygous expression of T118M PMP22 is disease predisposing but is incompletely penetrant and results in, at most, only mild peripheral neuropathy.

**Table 1:** Incidence of the T118M *PMP22* mutation in the human population (data extracted from gnomAD)

Population	Total *PMP22* alleles sequenced	Number of T118M *PMP22* alleles observed	Incidence in population
African/African American	24,934	21	1 in 594
Ashkenazi Jewish	10,534	48	1 in 110
East Asian	19,938	3	1 in 3323
European (excluding Finnish)	128,884	855	1 in 75
Finnish	25,014	156	1 in 80
Latino/Admixed American	35,428	16	1 in 1107
South Asian	30,616	39	1 in 392
Other	7212	37	1 in 97
Women	153,242	627	1 in 122
Men	129,138	548	1 in 118
All ethnicities/genders	282,380	1175	1 in 120

### The T118M PMP22 membrane protein traffics to the cell surface much less efficiently than WT

WT PMP22 is known to traffic to the plasma membrane (PM) with an efficiency of roughly 20% (23, 24, 25, 26, 27), whereas several disease mutants are known to be severely trafficking deficient (26, 27, 28, 29). For example, L16P PMP22, which causes DSS (severe neuropathy), traffics to the PM of Madin–Darby canine kidney (MDCK) cells with an efficiency of <2% (26). We here examined trafficking in cells expressing either WT or T118M PMP22, each in two different cell types (human embryonic kidney [HEK] and MDCK). In the trafficking assay (26, 27), cells transiently expressing myc-tagged PMP22 first have PM-localized protein labeled *via* immunofluorescence with a myc-specific antibody conjugated to a fluorophore. The myc tag is in the second extracellular loop, and only cell surface–localized protein is accessible to labeling. Cells are then permeabilized, and the remaining internal population of PMP22 is labeled with a myc-specific antibody conjugated to a different color fluorophore. Cells are then analyzed by flow cytometry, and the ratio of the normalized intensities of the two labels can be used to calculate the single cell surface trafficking efficiencies for thousands of cells, defined as the surface population divided by total (surface + internal) population × 100.

**Figure 2:**
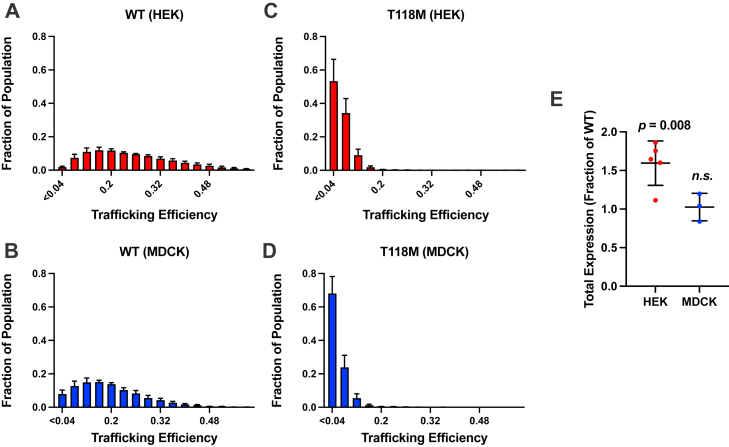
**Fractional trafficking efficiency and total expression of PMP22 WT and T118M.** Histograms of WT PMP22 trafficking efficiencies in human embryonic kidney (HEK) (*A*) and Madin–Darby canine kidney (MDCK) (*B*) cells. The trafficking efficiency is the level of PMP22 on the surface over total cellular PMP22 (surface + internal), as expressed in fractional units (to convert to percent, multiply by 100). Histograms of T118M trafficking efficiencies in HEK (*C*) and MDCK (*D*) cells. *E*, comparison of the total T118M PMP22 expression levels in HEK and MDCK cells (Mann–Whitney tests used to compare normalized T118M expression to WT for each cell type). For the HEK cell data, *n* = 5 experiments with 2500 cells each. For MDCK cell data, *n* = 3 experiments with 2500 cells each. PMP22, peripheral myelin protein 22.

T118M PMP22 was seen to exhibit a decreased fractional trafficking efficiency relative to WT in both HEK and MDCK cells (Fig. 2, *A*–*D*). In MDCK cells, WT PMP22 traffics with a 17 ± 3% efficiency, whereas T118M traffics with a 3.5 ± 1.1% efficiency. In HEK cells, WT PMP22 traffics with a 25 ± 5% efficiency, whereas T118M traffics with a 5.0 ± 1.6% efficiency. In both cell types, WT PMP22 shows a broad distribution of trafficking efficiencies across single cells (Fig. 2, *A* and *B*), whereas T118M has a much narrower range of efficiencies in single cells, indicating that the behavior of this mutant is more homogenous than WT. We next looked at total (surface + internal) expression for both WT and T118M PMP22 in HEK and MDCK cells. In HEK cells, total expression of T118M PMP22 is moderately higher than for WT, whereas total expression levels in MDCK cells were similar for WT and T118M (Fig. 2*E*). These results indicate that even though T118M PMP22 is more prone to mistraffic (*i.e.*, to be retained inside the cell rather than progressing to the PM) than WT PMP22, it is not more susceptible to degradation, at least following transient transfection in model cell lines after 48 h. Also, with an observed trafficking efficiency of 3.5 to 5% depending on the cell type, T118M PMP22 traffics slightly better to the PM than most other CMT mutant forms of PMP22 (26). Very likely, the mistrafficking of T118M PMP22 occurs only en route from the endoplasmic reticulum to the PM, rather than involving increased endocytosis or reduced endosome-to-PM recycling of the protein after initially reaching the PM. Given that in native peripheral nervous system tissue PMP22 is observed mainly in myelin membranes derived from the PM, it seems unlikely that it is subject to endocytosis. Indeed, endocytosis of PMP22 has not previously been documented, although it cannot be completely ruled out based on the currently available data.

**Figure 3:**
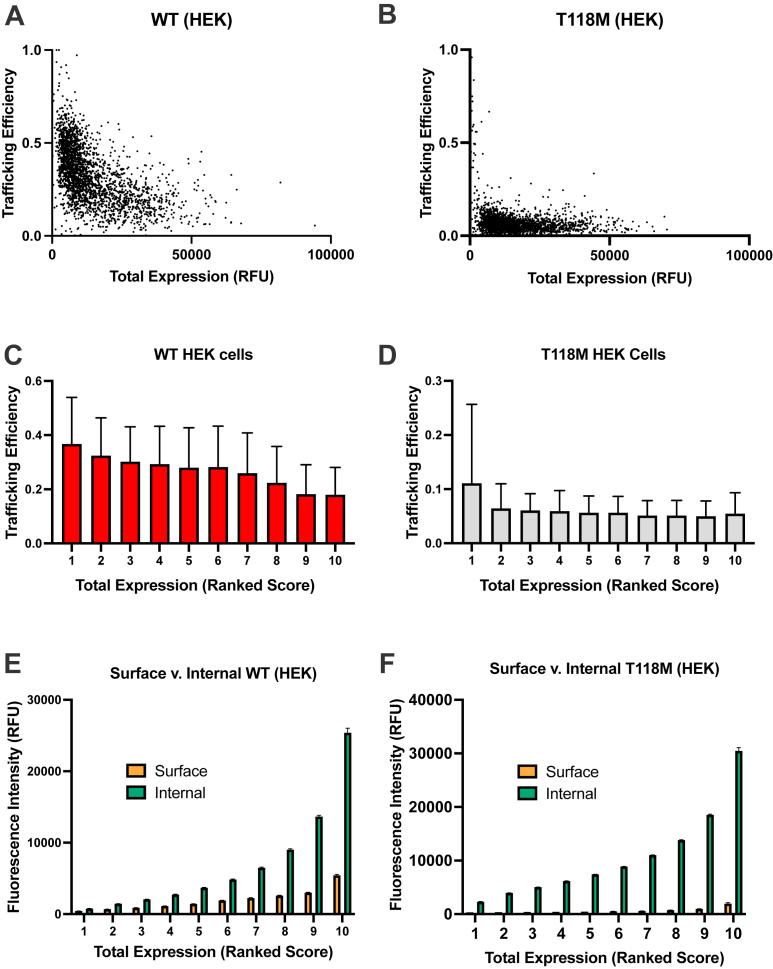
**The relationship between total expression and fractional trafficking efficiency differs between WT and T118M.** The data for WT in this figure were previously reported (23). The fractional trafficking efficiency is the level of PMP22 on the cell surface over total cellular PMP22 (surface + internal; to convert to percent units, multiply by 100). An inverse correlation is observed between trafficking efficiency and total expression for WT PMP22 that is less pronounced for T118M PMP22 (*A* and *B*) (data in *A* and *B* are from one representative experiment, 2500 single cells each). Trafficking efficiency data were binned from low expression (1) to high expression (10) for WT PMP22 and PMP22 T118M (*C* and *D*). WT shows consistent bin-to-bin changes that T118M does not (bars represent means ± SEM, *n* = 3). Relative fluorescence intensities of surface and internal PMP22 binned by ranked expression for WT and T118M (*E* and *F*) (bars represent means ± SD, *n* = 3). PMP22, peripheral myelin protein 22.

We used single-cell measurements to more closely examine the relationship between total expression and cell-surface trafficking. In HEK cells expressing WT PMP22, there is an inverse correlation between total cellular protein and trafficking efficiency (Fig. 3*A*, see also Ref. (23)). In other words, for cells that make more PMP22, a lower fraction of the total reaches the cell surface. This relationship, however, is weaker for T118M (Fig. 3*B*). Binning the data demonstrates a gradual bin-to-bin decrease in cellular trafficking efficiency with increasing total WT expression (Fig. 3*C*), whereas the only significant bin-to-bin change for T118M PMP22 is from the first to second bin, with the remaining bins exhibiting similar trafficking efficiencies (Fig. 3*D*). Examination of the raw fluorescence intensities from both surface and internally labeled PMP22 again shows a correlation between total expression and surface trafficking in WT (Fig. 3*E*) that is not seen for T118M (Fig. 3*F*). These data suggest that as higher cellular levels of WT PMP22 are expressed, its propensity to mistraffic increases. In the case of T118M, at all higher levels of expression (bins 2–10 in Fig. 3*D*), the trafficking efficiency is independent of total cellular expression level.

The aforementioned results indicate that while T118M exhibits a higher overall propensity to misfold and mistraffic, it is either less prone to form concentration-dependent aggregates than WT or that the folding defects in T118M are recognized earlier in the biosynthesis-to-cell surface pathway, leading to intracellular retention by quality control before aggregation has a chance to occur. We next conducted experiments to determine which of these possibilities actually pertains.

### Mistrafficked T118M PMP22 is less prone to form large aggregates than L16P PMP22

**Figure 4:**
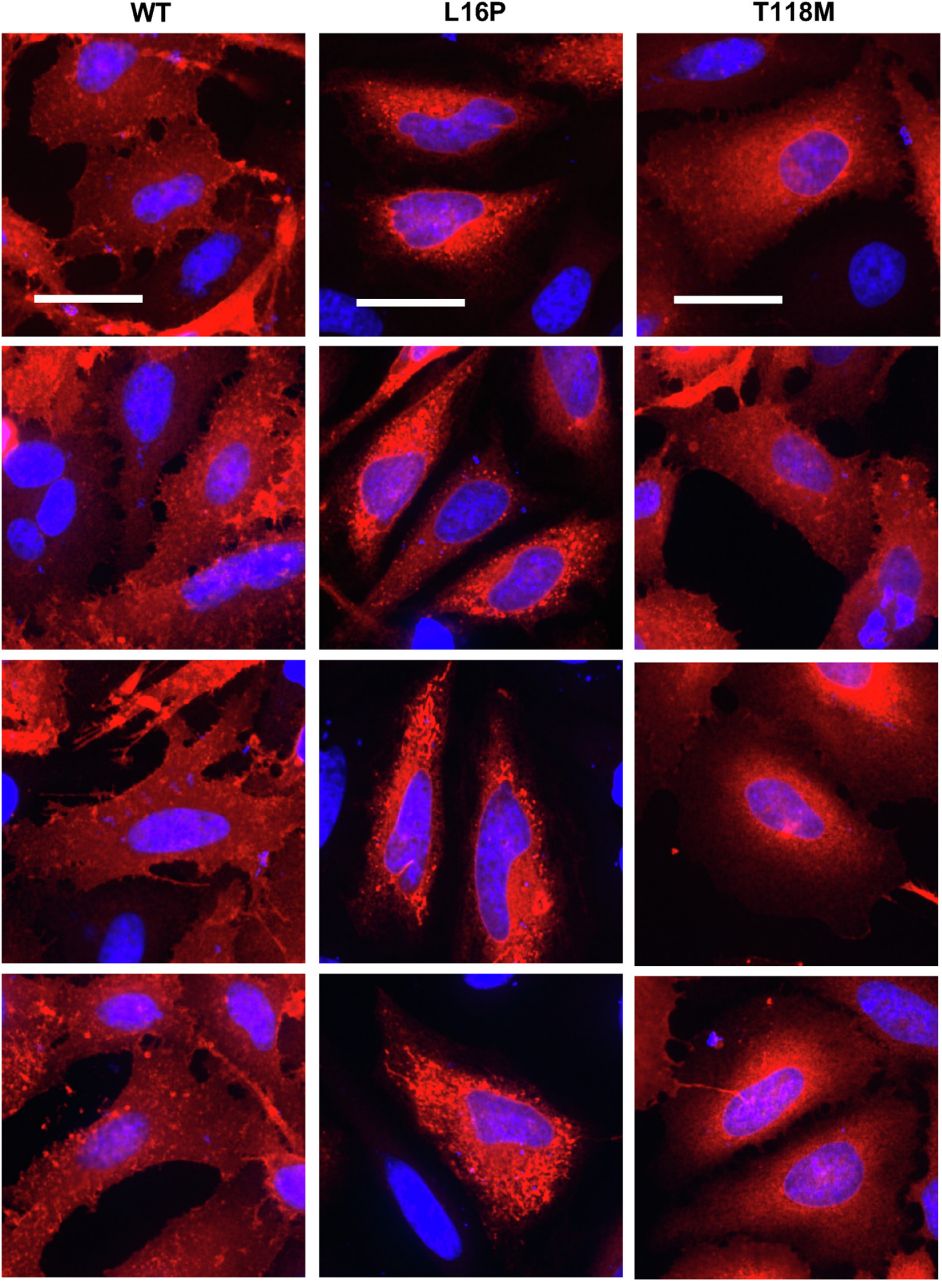
**L16P and WT PMP22 are more prone to form visible intracellular aggregates than T118M PMP22.** Distribution of PMP22 WT, L16P, and T118M in HeLa cells. Cells expressing PMP22 variants (WT, L16P, or T118M) for 48 h were fixed. PMP22 was labeled *via* immunofluorescence (*red*), and nuclei were labeled with DAPI (*blue*). Four representative fields of view (cropped and enlarged from 60× magnification) for each variant are shown. Cells expressing L16P exhibit multiple large intracellular foci. The same observation can be made to a lesser degree for WT PMP22, but T118M exhibits a more diffuse distribution. Scale bars represent 30 μm, scale is identical for all images. Images are representative from three independent biological replicates. DAPI, 4′,6-diamidino-2-phenylindole; PMP22, peripheral myelin protein 22.

We undertook to compare the formation of intracellular aggregates by T118M PMP22 to WT PMP22 and L16P PMP22, the latter of which causes severe neuropathy (DSS). WT, T118M, and L16P PMP22 were expressed in HeLa cells, which were then fixed, permeabilized, and immunostained. Panels of representative confocal images for each of these three forms of PMP22 are shown in Figure 4. The pronounced intracellular localization of T118M and L16P PMP22 relative to the WT protein is evident in these images, as expected based on the trafficking assay results of this and previous studies (26, 29). Also evident is the high abundance of puncta in the L16P-expressing cells, reflective of increased formation of large intracellular aggregates by L16P PMP22. This is consistent with results from prior studies (30, 31, 32, 33, 34). These puncta appear to be more rare in T118M-expressing cells (Fig. 4).

**Figure 5:**
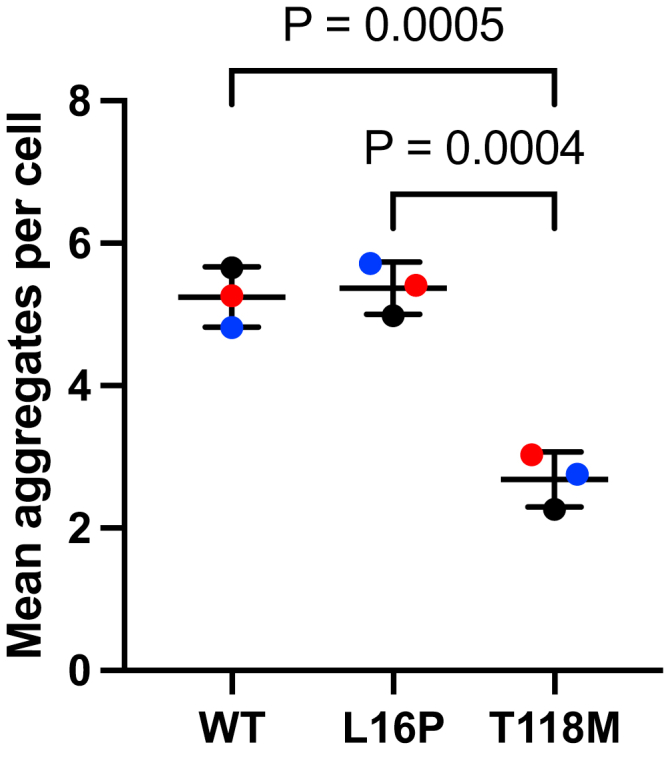
**T118M forms fewer intracellular aggregates than L16P or WT PMP22.** HeLa cells expressing PMP22 variants were imaged (as seen for Fig. 4). Intracellular aggregation from five representative images from each of three different biological replicates (15 images total per variant) were analyzed. The mean number of aggregates per cell for each biological replicate are shown. Bars represent mean ± SD. Symbol colors (*blue*, *red*, and *black*) represent the three biological replicates. *p* Values were generated from ANOVA and Tukey’s multiple comparisons tests. Single-image and single-cell quantifications are presented in Figs. S1 and S2. PMP22, peripheral myelin protein 22.

To more quantitatively compare puncta formation by T118M PMP22 *versus* WT and L16P forms of PMP22, we used the method of Klickstein *et al.* (35) to quantitate intracellular aggregate (puncta) formation by each form of PMP22 based on analysis of the immunofluorescence confocal microscopy images, as exemplified by Figure 4. The results are shown in Figure 5 and Fig. S1, where it was confirmed that T118M is less prone to form intracellular aggregates than L16P. Very notably, it is seen that the tendency of T118M to form aggregates is even lower than observed for WT PMP22. The single-cell results underlying Figure 5 are shown in Fig. S2 and are consistent with the notion that for WT, T118M, and L16P, the number of aggregates per cell correlates positively with expression. However, T118M forms fewer aggregates at all expression levels.

These results suggest that, while prone to mistraffic, T118M PMP22 induces only a mild form of CMT in part because it does not significantly form toxic intracellular aggregates.

### Folding and trafficking of WT PMP22 is unrelated to which codon is used to encode Thr118 in PMP22

**Figure 6:**
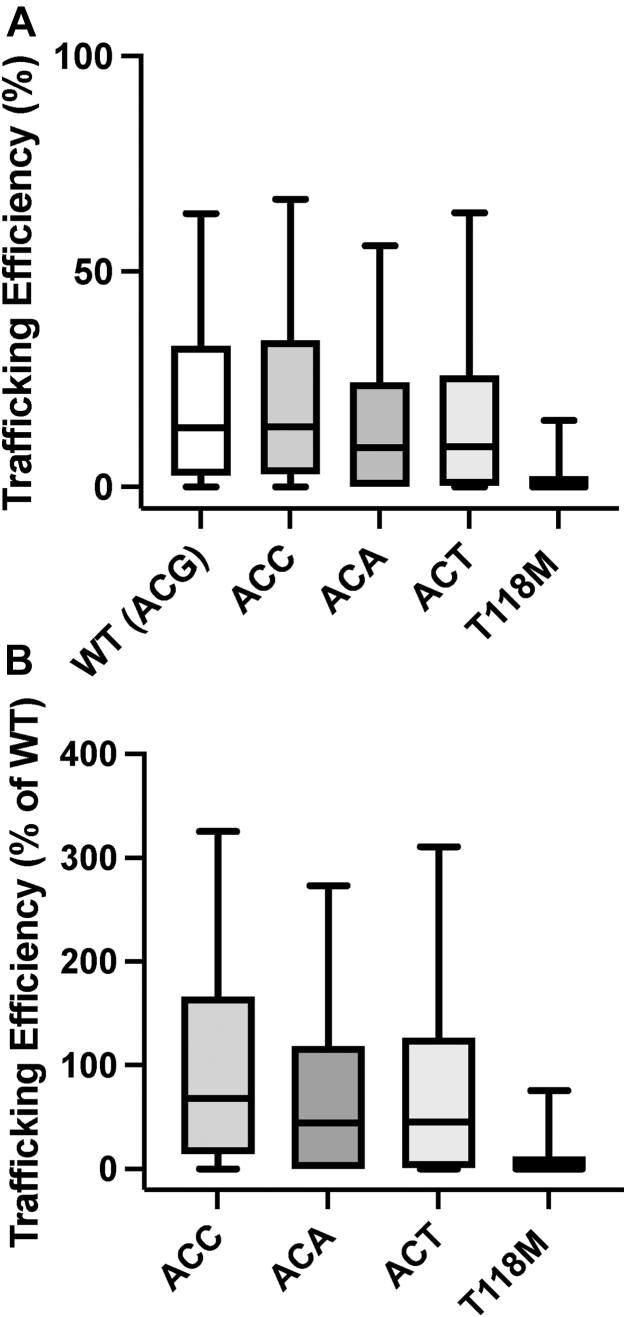
**Codon usage does not explain T118M PMP22 trafficking deficiency.***A*, presents the absolute trafficking efficiency (surface PMP22 relative to total PMP22), whereas *B* presents the percent of surface trafficking of each silent mutation variant relative to WT PMP22 with its WT T118 codon. Trafficking efficiencies for alternate silent Thr-118 codon mutants show similar trafficking efficiencies to the WT gene (*A* and *B*) (box and whisker plots shown, whiskers range from 5th to 95th percentiles, *n* = 2–3 experiments with 2500 cells each). PMP22, peripheral myelin protein 22.

We sought additional insight into why T118M results in deficient trafficking. WT T118 is encoded by ACG, a rare codon in humans. In contrast, the ATG codon that encodes Met in the T118M variant is a common codon. tRNAs that recognize rare codons are less abundant in cells, which leads to ribosomal pausing at these codons during translation (36, 37). We wondered whether ribosomal pausing at the T118-encoding codon during translation is important for PMP22 to adopt its correct fold. To test this, we used site-directed mutagenesis to generate three silent mutations in WT PMP22 to the other three possible threonine codons for site 118. We hypothesized that if lack of ribosomal pausing results in the observed trafficking defects for the T118M mutant, then by silently switching the rare WT T118 codon to a common Thr codon, the same defect in trafficking would be observed even without mutating Thr118. We tested the trafficking efficiency of each silent-118 mutant form of WT PMP22 in HEK cells using our flow cytometry assay. In these cells, T118M traffics with roughly 30% of the efficiency of WT (5.9 ± 4.3% for T118M *versus* 18 ± 8% for WT, Fig. 6). We did not observe dramatic changes in trafficking efficiencies for the three silent-118 mutation forms of WT (18 ± 11% for the ACC codon, 13 ± 1% for the ACA codon, and 15 ± 3% for the ACT codon, Fig. 6). From these results, we conclude that ribosomal pausing at T118 is not necessary for WT-like efficiency of PMP22 trafficking to the PM.

### Coexpression of T118M and WT PMP22 does not result in a significant dominant negative impact of T118M PMP22 coexpression on WT PMP22 trafficking

**Figure 7:**
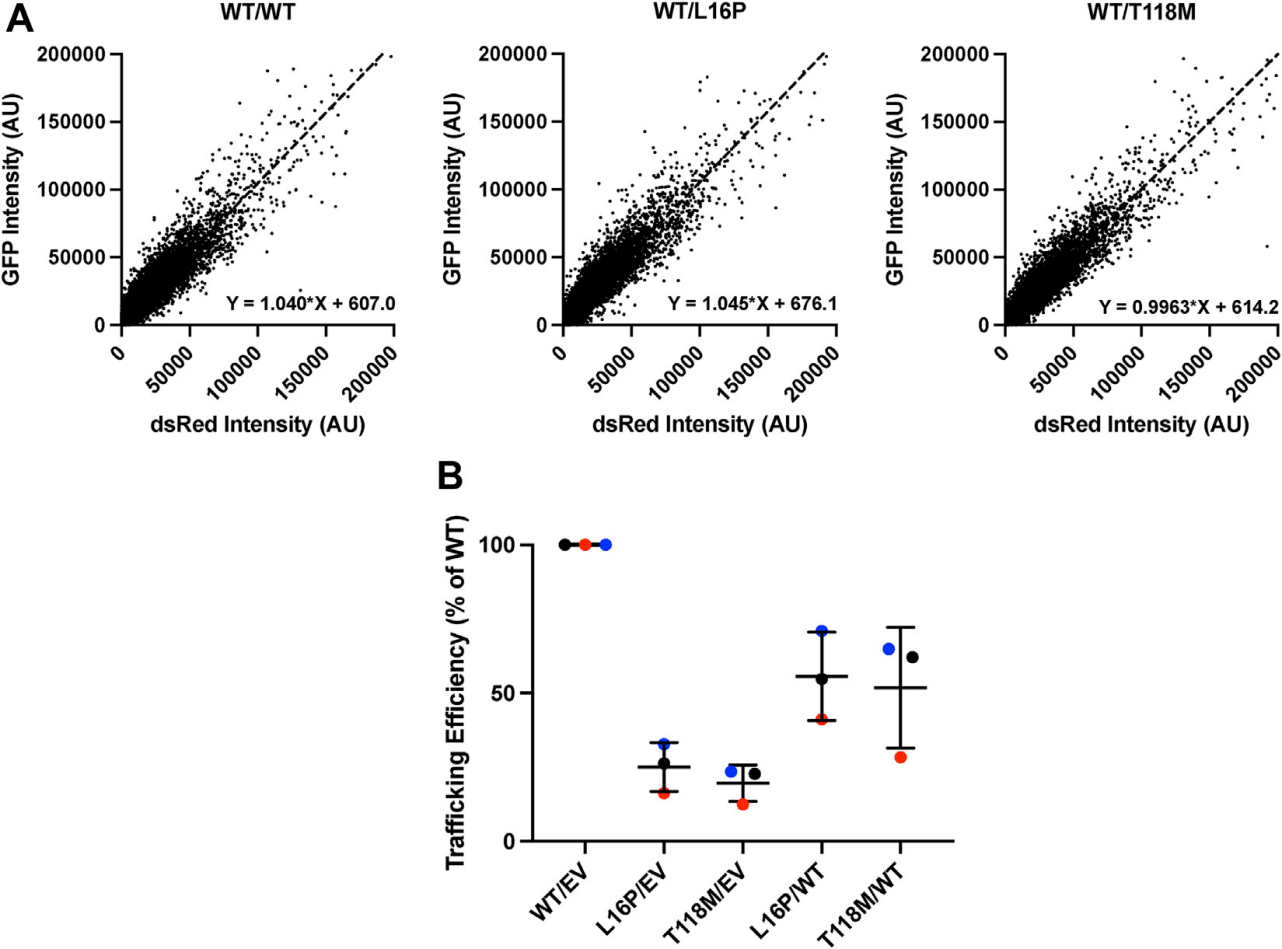
**Impact of coexpressing WT and PMP22 variants on total surface PMP22 levels.***A*, human embryonic kidney (HEK) cells cotransfected with two plasmids express equivalent amounts of protein from each plasmid. Cells were cotransfected with pIRES PMP22 dsRED (WT) and pIRES PMP22 enhanced GFP (eGFP) (WT, L16P, or T118M) and then analyzed by flow cytometry. The brightness-corrected intensities of the eGFP and dsRED from 50,000 cells (*n* = 1) each are shown. A slope of ∼1 indicates that cells express the protein from the two plasmids in a 1:1 ratio. *B*, relative trafficking efficiencies of PMP22 T118M and L16P cotransfected with WT PMP22. Trafficking efficiencies relative to WT are shown from cells cotransfected with WT, L16P, or T118M PMP22 and either WT PMP22 or an empty vector (EV) control. “Relative” means the reported efficiencies are normalized to the trafficking efficiency of WT PMP22 alone. Points are from three independent replicates (biological replicates are grouped by color) each with 2500 cells, bars represent means ± SD. PMP22, peripheral myelin protein 22.

We next hypothesized that in a heterozygous (WT/mutant), genetic background expression of the WT *PMP22* allele might partially rescue the trafficking of T118M, resulting in increased total trafficking efficiency in Schwann cells relative to the T118M-only expression conditions of the assays described in the previous sections. To test this, we conducted trafficking assays in which we cotransfected WT and either L16P or T118M PMP22 expression vectors in HEK cells at a ∼1:1 ratio (Fig. 7*A*). Cotransfection resulted in similar levels of total protein expression across all variants (Fig. S3). Coexpression of T118M and WT PMP22 resulted in a total PMP22 trafficking efficiency that is 52 ± 20% relative to WT-only trafficking (Fig. 7*B*). Although this is a little lower than the 60% average between WT-only expression (normalized to 100%) and Thr118M-only (20 ± 6% of WT), the WT/T118M data indicate that there is not a strong interaction between T118M and WT that alters trafficking of either protein. In other words, trafficking of T118M is not significantly “rescued” by coexpression with WT PMP22; conversely, mistrafficking of WT is not significantly induced by coexpression with T118M. Similar results were obtained for coexpression of L16P PMP22 with WT (Fig. 7*B*). These results disprove our hypothesis, instead suggesting that the mild nature of the phenotype associated with symptomatic WT/T118M heterozygous individuals is related to the fact that considerable PMP22 still correctly surface traffics in these human subjects—most from expression of the WT allele, but with a significant contribution from T118M. In this regard, the situation is akin to the WT/null hemizygous conditions resulting in HNPP, a disorder that also is mild. Indeed, it is known that the phenotype of CMT caused by heterozygous expression of T118M PMP22 is similar in symptoms to HNPP (16, 18), as expected given that the total amount of PMP22 that reaches the cell surface under WT/null conditions is similar to that occurring under WT/T118M conditions.

### NMR data indicate that the structure and dynamics of folded T118M PMP22 resemble WT PMP22

**Figure 8:**
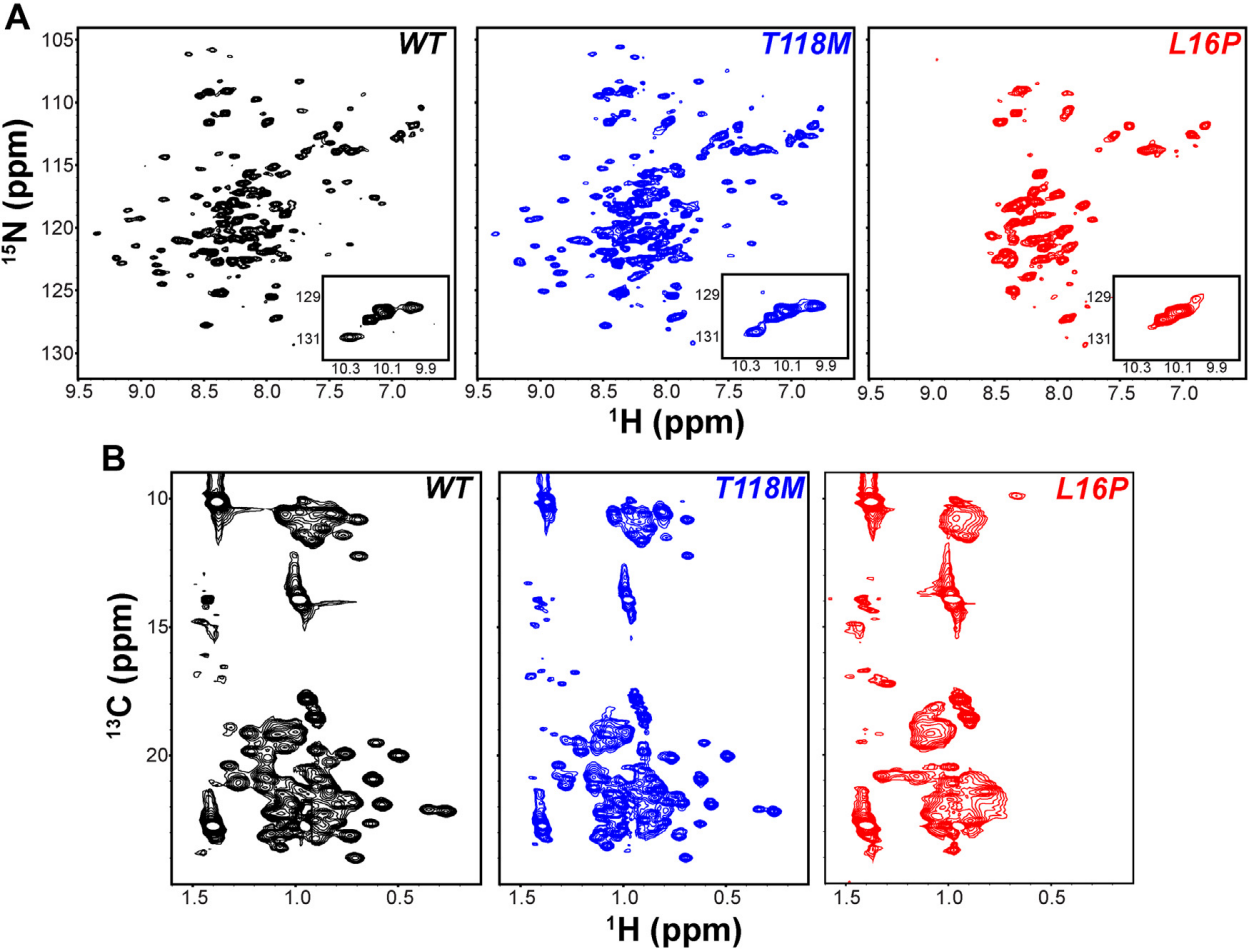
**NMR spectra of WT and variant forms of PMP22.***A*, ^1^H–^15^N-TROSY and (*B*) ^1^H–^13^C methyl TROSY spectra comparisons of WT, T118M, and L16P U-^15^N-PMP22 in DDM micelles. The samples were prepared in 20 mM acetate (pH 5.0), 100 mM NaCl, 5 mM TCEP, and 1 mM EDTA. The spectra were acquired at 45 °C using an 800 MHz spectrometer. DDM, dodecylmaltoside; HSQC, heteronuclear single quantum coherence; PMP22, peripheral myelin protein 22; TCEP, Tris(2-carboxyethyl)phosphine; TROSY, transverse relaxation optimized spectroscopy.

To qualitatively assess the structure of T118M PMP22 as compared with WT, we acquired the ^1^H–^15^N backbone amide and ^1^H–^13^C methyl side chain (Ile, Leu, and Val) transverse relaxation optimized spectroscopy (TROSY) NMR spectra of WT, T118M, and L16P PMP22 (Fig. 8). Both the amide and methyl TROSY spectra of T118M in dodecylmaltoside (DDM) micelles showed well-dispersed peaks with chemical shifts similar to those seen for the WT protein. This is in contrast to the severe disease-causing L16P mutant, which exhibited poorly dispersed amide and methyl TROSY spectra (Fig. 8), reflecting its structural instability. This indicates that T118M PMP22 maintains the same folded state under DDM micelle conditions as the WT protein. While less stable than WT PMP22 (26), its folding equilibrium still favors a WT-like folded state in DDM, unlike the even more unstable L16P.

**Figure 9:**
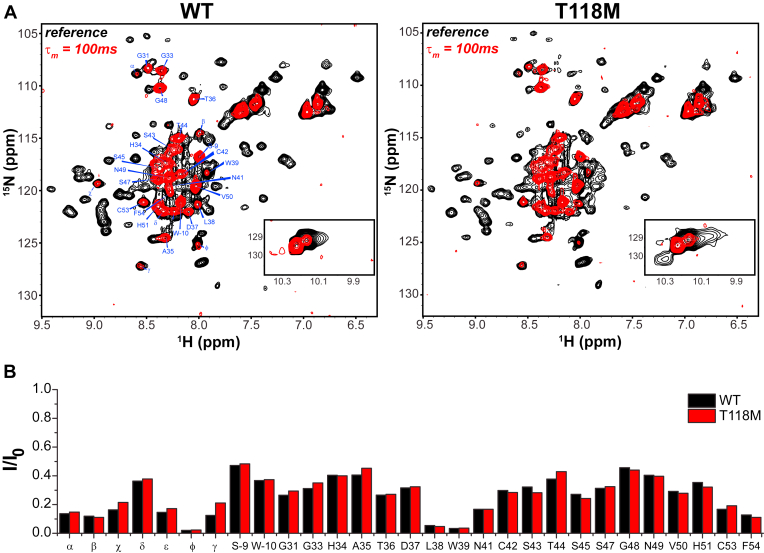
**Comparison of backbone amide proton exchange for T118M and WT.***A*, CLEANEX-PM HSQC spectra (100 ms mixing time, in *red*) superimposed on reference HSQC spectra (in *black*) for both WT and T118M. *B*, CLEANEX-PM intensity ratios (I/I_0_) comparison between WT and T118M. I is the intensity of the peak at 100 ms mixing time, whereas I_0_ is the intensity of the peak in the reference spectrum. The intensities of the 100 ms mixing time peaks indicate their susceptibility to exchange (*red peaks* are seen for sites where exchange is fast on the 100 ms time scale). The limited peak assignments shown are tentative and based on previously reported spectral assignments in tetradecylphosphocholine micelles (40). The samples were prepared in 20 mM PIPES (pH 6.5), 100 mM NaCl, 5 mM TCEP, 1 mM EDTA, and ∼6% DDM. DDM, dodecylmaltoside; HSQC, heteronuclear single quantum coherence.

To probe for possible differences in dynamics between T118M and WT PMP22, we measured backbone amide hydrogen exchange rates using the CLEANEX-PM (38, 39) NMR experiment. The CLEANEX-PM heteronuclear single quantum coherence (HSQC) with a 100 ms mixing time is compared with the reference HSQC with no mixing time in Figure 9*A*. We observed 29 backbone amide peaks that rapidly exchange with water in both T118M and WT (Fig. 9). Examining the peaks in light of previous partial resonance assignments for PMP22 (40), we identified that the rapidly exchanging peaks are mainly coming from the first extracellular loop of the protein (Fig. 9). When the intensity ratios were analyzed, we did not see any differences in the backbone amide hydrogen exchange rates between the two forms of the protein (Fig. 9*B*). We note that PMP22 is known to be a very slow folding protein (41), and so it is not surprising that the difference in folding stability between WT and T118M PMP22 was not manifest in the exchange rates.

These results indicate that the overall dynamics of T118M is similar to WT. Unlike the L16P mutant form of PMP22, the T118M mutant exhibits similar structure and dynamics to the WT protein, at least under the micellar conditions of these experiments.

### The T118M PMP22 allele predisposes human subjects to chronic or repeated CTS

To probe for additional and previously undetected roles for T118M *PMP22* on human health, we searched records for 88,308 human patients with genotyping data available in BioVU (42). BioVU is a DNA biobank that is combined with deidentified patient electronic medical records (EMRs) (Table S1). This search led to identification of 816 heterozygous carriers and ten homozygous carriers of the T118M variant (rs104894619). The population frequency of the T118M variant in this 88K patient cohort was 0.93%. This is similar to the frequency of 0.83% observed for this allele in the overall US population (see aforementioned section). We next searched for disorders outside the CMT family that are linked to this gene variation.

As a common peripheral neuropathy that is not classified in the CMT family, we tested for association of T118M *PMP22* with CTS. Our test cohort contained 3225 patients with a record of CTS (identified by the presence of International Classification of Disease (ICD) codes for CTS as described in the Analysis of the BioVU repository methods section). We carried out a logistic regression for the association of the T118M allele with CTS case status, adjusting for sex, age, duration of medical record, and using the first five genetic principal components to represent population substructure. We found that the T118M allele was not significantly associated with CTS case status (odds ratio [95% confidence interval] = 1.3 [0.9–1.8], *p* = 0.15, Table S2).

**Figure 10:**
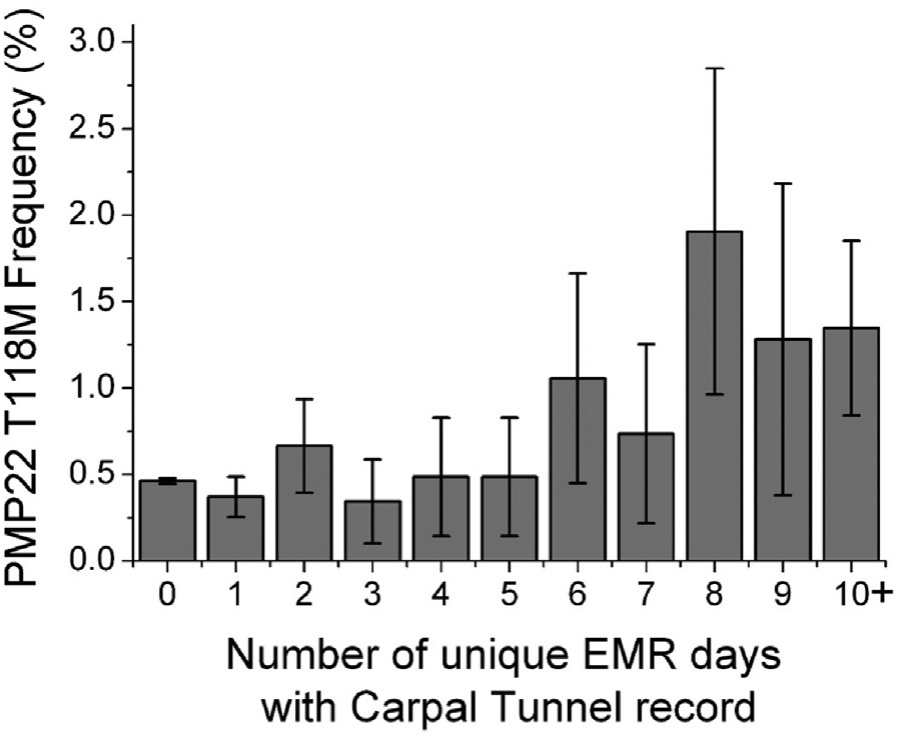
**PMP22 T118M allele frequency for deidentified patients with increasing length of medical record of carpal tunnel syndrome.** Error bars represent the standard error of sample proportion. EMR, electronic medical record; PMP22, peripheral myelin protein 22.

While the T118M gene variation was seen not to be predictive for CTS, we also probed whether this variation might predispose patients to long and/or repeated episodes of CTS—a measure of the degree of disease severity. One strength of BioVU is that it provides a longitudinal record extending over approximately 25 years. To quantify chronic CTS, we determined the total number of unique days with an EMR of CTS for the 3225 cases (Fig. 10). These days may either be scattered over many years, indicating repeated episodes of CTS, or may reflect long episodes indicating a persistent case. We carried out a linear regression for the total number of recorded CTS days and the T118M allele, with the same covariates as aforementioned. The T118M variant was strongly associated with the number of days in the medical record with a CTS ICD code (regression beta = 0.13 ± 0.07, *p* = 0.00032, Table S3). The regression beta is positive, indicating that carriers of the T118M variant on average have more days with the CTS code. The upper quintile of the distribution of the number of days with CTS record was 7 or more days, and we took that as a simple definition of chronic CTS. We carried out a logistic regression of these 579 chronic CTS cases *versus* controls (excluding all CTS cases with less than 7 days of record), adjusted for the same covariates as aforementioned. The T118M allele was significantly associated with chronic CTS (odds ratio [95% confidence interval] = 2.7 [1.6–4.6], *p* = 0.00018, Table S4). This result links T118M as a risk factor mutation for the chronic form of a well-known disorder outside the CMT family of peripheral neuropathies.

Our results establish the T118M-encoding *PMP22* mutation as a risk factor variant for chronic or repeated episodes of CTS. This is in contrast with other known CMT *PMP22* variants, which are thought to be 100% penetrant as a cause of CMT, even under WT/mutant heterozygous conditions.

## Discussion

The recognition that the incidence of the T118M PMP22 allele in the general population is roughly 20 times more common than the total incidence of all forms of CMT clarifies the relationship to CMT of this variant allele under heterozygous conditions. Namely, T118M PMP22 is a risk factor that predisposes human subjects to a typically mild form of CMT, with a penetrance of <5%. It is not clear from this study what the colluding risk and/or protective factors are that determine which T118M PMP22 carriers develop CMT.

T118M PMP22 carriers who do present with CMT typically suffer from only mild peripheral neuropathy, similar to PMP22 haploinsufficient (WT/null) HNPP patients. This study presents new data leading to a rather thorough understanding of the molecular and cellular properties of the T118M protein, to the extent that we can offer a model for how this variant predisposes patients to CMT and that explains why symptoms, when seen, are usually mild. It has previously been shown that T118M is less thermodynamically stable than WT PMP22 (26, 43). As confirmed in this work (Figs. 2 and 3), it has also been shown that the surface trafficking efficiency of WT and disease mutant forms of PMP22, including T118M, correlates linearly with the thermodynamic stability of the protein (26). This reflects the fact that the cellular membrane protein folding quality control system is attuned to be able to recognize unstable PMP22. Previous PMP22 trafficking studies have shown that knockout of either the chaperones calnexin or RER1 signficantly increases the forward trafficking efficiency of T118M PMP22 (Fig. S10 in Ref. (27)), suggesting these proteins may be involved in retention of this mutant early in the secretory pathway.

While less stable and more prone to mistraffick than WT, our results revealed that T118M PMP22 in DDM micelles has sufficient stability to remain folded in a conformation that resembles the WT protein. Its conformational dynamics were also seen to be similar to WT (Figs. 8 and 9). This highlights critical points of similarity between WT and T118M PMP22 that help explain why the T118M mutation is so mild in terms of pathogenic consequences. T118M differs from the L16P form of PMP22, the latter of which was seen to be partly or completely unfolded in DDM micelles (Fig. 8). L16P was previously shown to have a specific mutation-caused conformational defect—a kinked transmembrane helix (40). It is possibly significant that the T118 site in PMP22 is atypical among most known disease mutation sites in this protein in that it is not involved in tertiary structural interactions located within the transmembrane domain but is instead located in the second water-exposed extracellular loop of PMP22 (Fig. 1).

The generally mild CMT phenotype associated with heterozygous expression of T118M suggests that reduced cell surface trafficking of a single PMP22 allele is insufficient to cause the more severe neuropathy and dysmyelination that is associated with both overexpressed WT PMP22 (CMT1A) and with a number of other disease mutant forms of PMP22, such as L16P (causing DSS or CMTE). Why is the T118M/WT PMP22 phenotype so mild?

T118M PMP22 differs from mutants such as L16P in that the latter is much more prone to form large intracellular aggregates, whereas T118M does not (Figs. 4 and 5, (26, 29, 30, 31, 32, 33, 34)). This suggests that the mild and incompletely penetrant disease phenotype caused by T118M reflects its failure to form toxic intracellular aggregates. Indeed, our data revealed that T118M is less prone to form intracellular aggregates even than WT PMP22. This is consistent with a previous study showing that overexpression of WT PMP22 under CMT1A conditions results in the aberrant accumulation of the WT protein in the late Golgi (44). Moreover, our data also suggest that T118M is different than WT PMP22 in that the trafficking efficiency of the WT protein is reduced as its total expression levels in cells increased, whereas for T118M PMP22, its trafficking efficiency, though low, is not reduced further as total cellular expression levels increase (Fig. 3, *C* and *D*). It may also be significant that T118M PMP22 was recently shown by ion mobility native mass spectrometry to be less prone to form dimers than WT PMP22, whereas L16P was seen to be much more likely to form misfolded dimers than WT (43). While highly mistrafficking-prone, T118M PMP22 has little tendency to form oligomers or aggregates and is evidently easier for cells to manage and degrade than more severe disease mutant forms, including WT PMP22 when overexpressed under CMT1A conditions.

The biophysical and/or cell biological basis for why the T118M mutation seems to protect PMP22 from forming intracellular aggregates is not established by our results but may be an interesting avenue for future investigation.

Based on the aforementioned considerations, we propose that the very mild form of CMT caused by T118M PMP22 is the consequence of reduced cell surface trafficking efficiency of the mutant, leading to reduced functional PMP22 at the cell surface. Relative to other PMP22 disease mutants and even WT, T118M PMP22 has reduced tendency to form visible intracellular aggregates. Moreover, its folded 3D structure and dynamics closely resemble the WT protein, such that the modest fraction of T118M that does reach the PM likely is functional. As a consequence, the CMT-class neuropathy caused by heterozygous WT/T118M PMP22 expression is similar to but even more mild than the HNPP phenotype seen for haploinsufficient (WT/null) patients.

A final conclusion of this article is that heterozygous expression of the T118M PMP22 variant is also a risk factor for chronic or repeated incidents of CTS. While not usually grouped with the CMT group of peripheral neuropathies, CTS does have some similarity to HNPP. For both disorders, symptoms are often triggered by nerve compression. Moreover, HNPP and CMT patients are also sometimes diagnosed with CTS (45, 46). This is the first study to quantitatively establish a genetic linkage between a PMP22 mutation and chronic CTS, indicating that CMT and CTS share at least one risk factor.

## Experimental procedures

### Cloning, cell culture, and transfection conditions

A myc epitope–tagged form of PMP22 was cloned into a pIRES2 backbone that coexpresses enhanced GFP or dsRed, as previously described (26). The myc epitope is located on the second extracellular loop and has previously been shown not to interfere with PMP22 cellular transport or function (28, 31). MDCK, HEK, and HeLa cells were cultured at 37 °C and 5% CO_2_ in F-12 Dulbecco's modified Eagle's medium (MDCK) or Dulbecco's modified Eagle's medium (HEK and HeLa) containing 10% fetal bovine serum, penicillin, and streptomycin (Life Technologies) in 6 cm culture dishes. MDCK cells were grown to 20 to 50% confluency and then transfected in OptiMEM media (Life Technologies) for 24 h with 1 μg of plasmid per dish using Effectine transfection reagent (Qiagen). The transfection media were then removed, and the cells were allowed to culture in complete growth medium for 24 h. HEK cells were cultured to 50 to 60% confluency and then transfected with 1.5 μg of plasmid using either a calcium phosphate–based methodology (Fig. 1, experiments) or Lipofectamine 3000 (Invitrogen) transfection reagent (Fig. 6, experiments). For cotransfections, 1.5 μg of total DNA was used with 0.75 μg of each variant or empty vector being premixed.

### PMP22 trafficking assay

Flow cytometry trafficking assays were conducted as described previously (26, 27). Briefly, cells were trypsinized and resuspended in culture media. Surface PMP22 was then labeled with a phycoerythrin-labeled anti–myc antibody (Cell Signaling Technology; catalog no.: 3739). Cells were then washed, fixed, and permeabilized (Invitrogen; catalog no.: GAS004). Then internal PMP22 was labeled with an anti–myc antibody conjugated to Alexa Fluor 647 (Cell Signaling Technology; catalog no.: 2233). Cells were then analyzed *via* flow cytometry on a BD LSRFortessa (Becton, Dickinson and Company). Cells expressing PMP22 were analyzed by gating on GFP-positive cells. Single-cell phycoerythrin and Alexa Fluor 647 values were collected. Intensities were corrected for nonspecific background and differences in intrinsic fluorophore brightness. Trafficking efficiencies were then calculated using the corrected surface (*i*_*surface*_) and internal (*i*_*internal*_) intensities as:

(1) $$Fractional\;trafficking\;efficiency=\frac{i_{surface}}{i_{surface}+i_{\mathrm{int}ernal}}$$

Histograms were generated by pooling 7500 cells per condition, which were then binned according to their trafficking efficiencies in bin sizes of 0.04 efficiency units.

### Quantitative immunofluorescence of PMP22 in cells

To observe the subcellular distribution of PMP22, immunofluorescence was conducted in HeLa cells. HeLa cells (ATCC; CCL-2) were seeded into 96-well plates (Greiner; catalog no.: 655090) at 5000 cells per well. One day after plating, cells were transfected with 50 ng of DNA per well using Fugene 6 (Promega) following the manufacturer’s protocol. pIRES PMP22-myc enhanced GFP WT, L16P, and T118M variants were used. About 48 h post-transfection, cells were fixed with 4% paraformaldehyde. After permeabilizing and blocking, cells were incubated with mouse anti–myc antibody (Cell Signaling; item 2276) followed by labeling with an antimouse secondary antibody conjugated to Alexa Fluor 647 (Cell Signaling; item 4410). The nuclei were subsequently labeled with 4′,6-diamidino-2-phenylindole. Plates were imaged on an ImageXpress Micro Confocal High Content Screening System (Molecular Devices) with a Nikon, 60× 0.95 numerical aperture Plan Apo Lambda Objectivean Andor Zyla 4.2MP 83% QE sCMOS camera, and an 89-North LDI five channel laser light source. Nine fields were imaged per well in an unbiased manner. The plate was imaged using a spinning disc confocal module with a 60 μm pinhole size. Images were collected using identical laser powers and acquisition times for all variants. Intracellular aggregates were quantified in ImageJ (US National Institutes of Health) using the AggreCount v1.13 macro (35). Five representative images from each of three biological replicates with moderate cell density were selected for analysis. All images were quantified with identical intensity thresholds, minimum and maximum aggregate sizes, and cell body segmentation strictness. Statistical comparisons were made in GraphPad Prism 9 (GraphPad Software, Inc).

### Expression and purification of PMP22

PMP22 was prepared as a fusion protein with the first 76 residues of the lambda repressor at the N terminus followed by a decahistidine tag, a thrombin cleavage site, a strep tag, and then the human PMP22 sequence. WT and T118M PMP22 were expressed in *Escherichia coli* and purified according to the previously reported protocols (26, 41) with some minor modifications. Briefly, the plasmid containing the PMP22 gene was transformed into BL21(DE3) star cells, and the bacteria were cultured in minimal media (M9) supplemented with ^15^NH_4_Cl at 20 °C. To label the methyl groups of the Ile, Leu, and Val residues, 70 mg of α-ketobutyric acid (methyl-^13^C) and 95 mg of α-ketoisovaleric acid (dimethyl-^13^C_2_) were added per liter of minimal media 1 h prior to induction (47). IPTG was added to induce protein expression into the M9 medium at 20 °C to a final concentration of 1 mM when an absorbance at 600 nm reached 0.8 to 1.0. Cells were harvested after ∼22 h and resuspended in the lysis buffer (75 mM Tris, 300 mM NaCl, 0.2 mM EDTA, pH 7.5).

Cell lysis and nickel–nitrilotriacetic acid (Ni–NTA) purification were carried out according to the previously published protocol (26, 41). Minor modifications in the protocol were implemented after the initial Ni–NTA purification of the fusion protein was carried out. The eluate from the first Ni–NTA purification containing the fusion protein in 50 mM Tris (pH 8.0), 500 mM imidazole, 1 mM Tris(2-carboxyethyl)phosphine (TCEP), and 0.25% DDM was buffer-exchanged twice with detergent-free 50 mM Tris (pH 8.0) and 1 mM TCEP before thrombin cleavage overnight at room temperature. The cleaved PMP22 (no longer containing a His_10_ tag) was separated from the uncleaved PMP22 by performing another Ni–NTA purification where the mixture was incubated with Ni–NTA resin for ∼2 h before transfer of the resin into a column and successive washing with 20 mM Tris buffer, 1 mM TCEP, and 0.05% DDM (pH 8.0) containing 20 mM, then 40 mM, and then 250 mM imidazole. The flow-through and eluate from the 20 mM imidazole washes were pooled together because they contained the cleaved PMP22. The pH of the mixture was then adjusted to 7.4 before incubating with 1 ml of Benzamidine Sepharose 4 Fast Flow High Sub (Cytiva) resin for 2 h to remove the thrombin. The flow through from this Benzamidine Sepharose equilibration was further purified by size-exclusion chromatography using a Superdex 200 Increase 10/300 GL (Cytiva) column with the NMR buffer (20 mM acetate [pH 5.0] or 20 mM PIPES [pH 6.5], 100 mM NaCl, 5 mM TCEP, 1 mM EDTA, and 0.2% DDM) as the mobile phase. Fractions containing pure PMP22 were pooled together and concentrated, followed by addition of D_2_O to 5% to prepare the final NMR samples.

### NMR spectroscopy of PMP22

Amide and methyl TROSY spectra were collected on samples containing 300 to 400 μM of either U-^15^N-labeled or Ile, Leu, and Val methyl-^13^CH_3_-labeled PMP22 (WT, T118M, or L16P) in DDM micelles. NMR spectra were acquired at 45 °C using an 800 MHz Bruker Avance III spectrometer equipped with a TCI cryoprobe.

Hydrogen exchange rates were measured for amide NH sites occurring during the timescale of the mixing time using the CLEAN chemical exchange experiment (CLEANEX-PM) with fast HSQC detection (39). The CLEANEX-PM experiment was carried out at 45 °C using a 100 ms mixing time on a sample containing 380 μM U–^15^N PMP22 (WT or T118M) in buffer containing 20 mM PIPES (pH 6.5), 100 mM NaCl, 5 mM TCEP, 1 mM EDTA, and ∼6% DDM. Peak intensities were compared with that of the reference HSQC spectrum collected with no mixing time. Both the CLEANEX-PM and the reference spectra were acquired using a 900 MHz Bruker Avance III spectrometer equipped with a TCI cryoprobe.

### Analysis of the BioVU biorepository and associated deidentified patient records for previously unrecognized T118M PMP22–disease relationships

BioVU is a large-scale DNA biobank representing ∼270,000 human subjects along with their deidentified EMRs. The work was determined by Institutional Review Board review to be nonhuman subjects research because of the completely deidentified nature of the biobank (Institutional Review Board #181443). Our cohort for this study consisted of 88,308 BioVU subjects previously genotyped on the Illumina MEGA-EX genotyping array, which includes the directly genotyped *PMP22* variant T118M (rs104894619). Table S1 gives the demographics of this cohort. Race and ethnicity categories in this table are those reported in the EMR.

CTS cases were defined by the presence of relevant ICD codes. The codes used in the ICD-9 system (used in the United States until October 1, 2015) were 354 (CTS) and 4.43 (release of carpal tunnel). Codes in the ICD-10 system (first used beginning October 1, 2015) were G56.00 (CTS, unspecified upper limb), G56.01 (CTS, right upper limb), G56.02 (CTS, left upper limb), and G56.03 (CTS, bilateral upper limbs). The presence of any of these ICD codes was used to define a carpal tunnel case. Controls were defined by the absence of all these ICD codes. For the cases, the total number of unique days with a carpal tunnel ICD code was recorded, and those cases with ≥7 days of EMR of CTS were noted as a separate class for analysis.

All statistics were calculated in R, version 3.6.3. Logistic regression was used for case–control status, and linear regression was used for ordinal and continuous variables. All regressions were adjusted for sex (coded as F = 0, M = 1), age at end of medical record, duration of medical record, and the first five genetic principal components.

## Data availability

All data needed to evaluate the conclusions in the article and supporting information are presented in the article or in the supporting information. Correspondence and requests for materials should be addressed to either DCS (david.c.samuels@vanderbilt.edu) or CRS (chuck.sanders@vanderbilt.edu).

## Supporting information

This article contains supporting information.

## Conflict of interest

The authors declare that they have no conflicts of interest with the contents of this article.
