# Supplementary material for: How T118M peripheral myelin protein 22 predisposes humans to Charcot–Marie–Tooth disease
Source: J Biol Chem. 2022 Dec 26;299(2):102839. doi: 10.1016/j.jbc.2022.102839 (PMC9860121; doi:10.1016/j.jbc.2022.102839)
Supplement: Supporting Figures S1–S3 and Tables S1–S4 [file mmc1.pdf]

*Supporting Information for*

# How T118M peripheral myelin protein 22 predisposes humans to Charcot-Marie-Tooth disease

Katherine M. Stefanski[1,2*], Geoffrey C. Li[1,2*], Justin T. Marinko[1,2],

Bruce D. Carter[1], David C. Samuels[3], and Charles R. Sanders[1,2,4]

[1]*Department of Biochemistry, Vanderbilt University School of Medicine, Nashville, TN 37240, USA*

[2]*Center for Structural Biology, Vanderbilt University School of Medicine, Nashville, TN 37240, USA*

[3]*Department of Molecular Physiology and Biophysics, Vanderbilt University School of Medicine, Nashville, TN 37240, USA*

[4]*Department of Medicine, Vanderbilt University School of Medicine, Nashville, TN 37240, USA*

*GCL and KMS contributed equally to this work

Correspondence should be addressed to: David C. Samuels (david.c.samuels@vanderbilt.edu) and Charles R. Sanders (chuck.sanders@vanderbilt.edu)

**This PDF file includes:**

Table S1
Table S2
Table S3
Table S4
Figure S1
Figure S2
Figure S3

**TABLE S1: Demographics of the BioVU cohort used in this analysis.**

| | Total cohort | Carpal Tunnel controls | Carpal Tunnel cases | Cases with Carpal Tunnel ICD on 7+ days |
|---|---|---|---|---|
| **Sample size** | 88,308 | 85,083 | 3,225 | 579 |
| **Female (%)** | 50,804 (58%) | 48,610 (57%) | 2,194 (68%) | 397 (69%) |
| **Caucasian** | 64,118 (73%) | 61,542 (72%) | 2,576 (80%) | 449 (78%) |
| **African American** | 14,108 (16%) | 13,623 (16%) | 485 (15%) | 99 (17%) |
| **Hispanic** | 2,586 (3%) | 2,528 (3%) | 58 (2%) | 12 (2%) |
| **Unknown Race/ Ethnicity** | 5,779 (7%) | 5727 (7%) | 52 (2%) | 12 (2%) |
| **Other Race/Ethnicity** | 1,717 (2%) | 1,663 (2%) | 54 (2%) | 7 (1%) |
| **Age, years*** | 53 [31-67] | 52 [30-66] | 62 [52-71] | 63 [55-72] |
| **Duration of Medical Record, years*** | 7.6 [2.7-13.2] | 7.3 [2.5-12.9] | 13.8 [9.3-17.6] | 15.1 [11.0-19.1] |
| **PMP22 T118M frequency** | 0.47% | 0.46% | 0.62% | 1.29% |

*Median [IQR}

**Table S2: Logistic regression results for carpal tunnel case status.**

| Variable | Regression beta | Std Err | Z value | P value |
|---|---|---|---|---|
| T118M | 0.237802 | 0.166868 | 1.425 | 0.15413 |
| Sex (M=1) | -0.45141 | 0.039276 | -11.493 | < 2e-16 |
| Age (years) | 0.025562 | 0.001013 | 25.232 | < 2e-16 |
| EMR duration (years) | 0.118734 | 0.003084 | 38.501 | < 2e-16 |
| PC1 | -0.05052 | 0.019291 | -2.619 | 0.00882 |
| PC2 | -0.03318 | 0.026316 | -1.261 | 0.20737 |
| PC3 | 0.003267 | 0.027056 | 0.121 | 0.9039 |
| PC4 | -0.00743 | 0.018818 | -0.395 | 0.69306 |
| PC5 | -0.00086 | 0.02072 | -0.041 | 0.967 |

**Table S3: Linear regression results of the number of unique days with a carpal tunnel ICD code.**

| Variable | Regression beta | Std Err | Z value | P value |
|---|---|---|---|---|
| T118M | 0.1273762 | 0.0354233 | 3.596 | 0.000324 |
| Sex (M=1) | -0.0590476 | 0.0069603 | -8.483 | < 2e-16 |
| Age (years) | 0.0029053 | 0.0001582 | 18.37 | < 2e-16 |
| EMR duration (years) | 0.0184093 | 0.0005619 | 32.762 | < 2e-16 |
| PC1 | -0.0126795 | 0.003502 | -3.621 | 0.000294 |
| PC2 | 0.0013152 | 0.0034909 | 0.377 | 0.70636 |
| PC3 | -0.0042312 | 0.003443 | -1.229 | 0.219097 |
| PC4 | -0.0035791 | 0.0034519 | -1.037 | 0.299801 |
| PC5 | 0.0010737 | 0.0034448 | 0.312 | 0.75527 |

**Table S4: Logistic regression results of chronic carpal tunnel syndrome cases (defined as 7+ days of record of carpal tunnel ICD codes).**

| Variable | Regression beta | Std Err | Z value | P value |
|---|---|---|---|---|
| T118M | 1.000242 | 0.267215 | 3.743 | 0.000182 |
| Sex (M=1) | -0.450126 | 0.090751 | -4.96 | 7.05E-07 |
| Age (years) | 0.030445 | 0.002423 | 12.563 | < 2e-16 |
| EMR duration (years) | 0.1556 | 0.007591 | 20.497 | < 2e-16 |
| PC1 | -0.133129 | 0.042263 | -3.15 | 0.001633 |
| PC2 | -0.057979 | 0.075169 | -0.771 | 0.440519 |
| PC3 | -0.142492 | 0.070518 | -2.021 | 0.043316 |
| PC4 | -0.03365 | 0.043407 | -0.775 | 0.438207 |
| PC5 | -0.013698 | 0.050967 | -0.269 | 0.788115 |

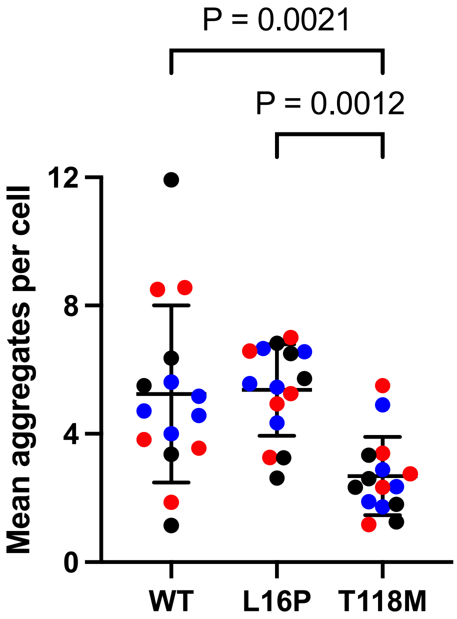


**Figure S1.** T118M forms fewer intracellular aggregates than WT and L16P. Data shown are mean aggregates per cell from single images (15 each from 3 biological replicates) as shown in Figure 5. Bars are mean ± SD. Points are grouped by color for the 3 biological replicates (blue, red, and black). P values were generated from ANOVA and Tukey's multiple comparisons tests.

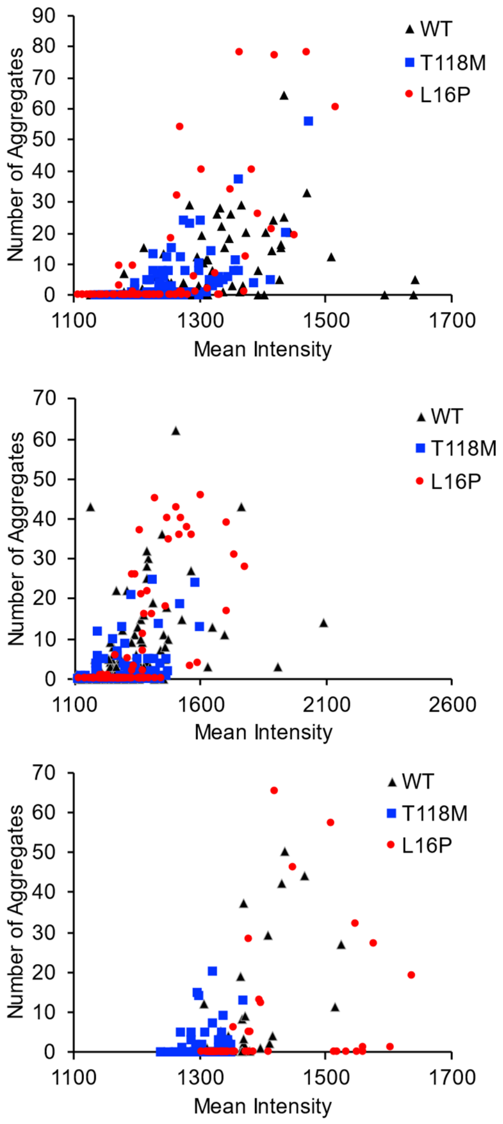


**Figure S2. Number of PMP22 aggregates vs amount of PMP22 per cell.** Data are from intracellular aggregate image quantification (as seen in Figures 4 and 5). Each point reports single cell quantification of the number of aggregates and the mean fluorescence intensity of immunolabeled PMP22, a proxy for expression levels. Each panel is an independent biological replicate.

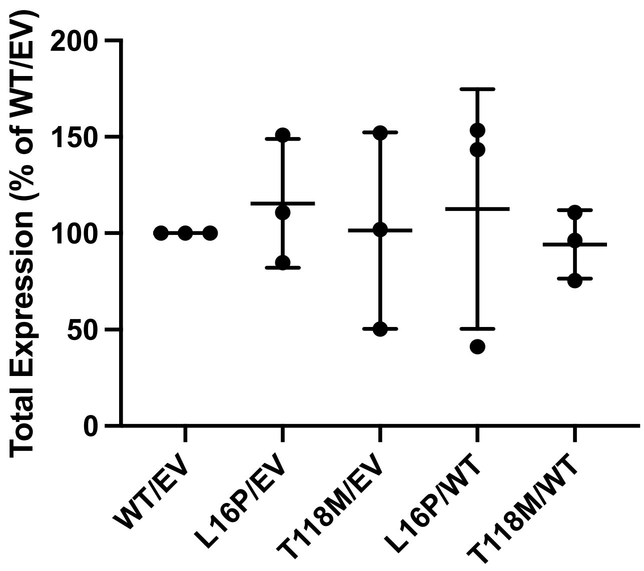


**Figure S3.** Total expression of PMP22 in co-transfected cells. No significant differences in expression were observed as determined by a Kruskal-Wallis test (P = 0.9309). Data are from trafficking assays shown in Figure 7.
